# Association between Physical Exercise and Biomarkers of Oxidative Stress among Middle-Aged and Elderly Community Residents with Essential Hypertension in China

**DOI:** 10.1155/2018/4135104

**Published:** 2018-07-03

**Authors:** Ying Yu, Qin Gao, Wanning Xia, Lina Zhang, Zhiyuan Hu, Xuesen Wu, Xianjie Jia

**Affiliations:** ^1^Department of Physiology, Bengbu Medical College, 2600 Dong Hai Avenue, Bengbu 233030, China; ^2^Science Research Center, Bengbu Medical College, 2600 Dong Hai Avenue, Bengbu 233030, China; ^3^Department of Epidemiology and Statistics, Bengbu Medical College, 2600 Dong Hai Avenue, Bengbu 233030, China; ^4^Department of Neurology, First Affiliated Hospital of Bengbu Medical College, Bengbu 233004, China

## Abstract

This study aimed to investigate the role of different types and frequencies of physical exercise in biomarkers of oxidative stress among middle-aged and elderly community residents with essential hypertension in China. A community-based cross-sectional survey was undertaken in 7 subdistricts. Individuals, 45-79 years old, with essential hypertension (*n* = 402) and without cardiovascular disease (*n* = 1047) were included. Superoxide dismutase (SOD) activities and plasma levels of malondialdehyde (MDA) and 4-hydroxynonenal (4-HNE) were determined. Multilevel linear regression was used to estimate the associations between various types of physical exercise and oxidative stress biomarker levels. Participants engaged in high frequency walking/square dancing or taiji/yoga demonstrated decreased systolic blood pressure in both groups; however, diastolic blood pressure decreased only among individuals with hypertension participating in walking/square dancing. In individuals with hypertension, MDA levels decreased in those participating in walking/square dancing, SOD activity increased in those participating in walking/square dancing, and 4-HNE levels decreased in those involved in taiji/yoga. In individuals without cardiovascular disease, MDA levels decreased in those involved in walking/square dancing or taiji/yoga, SOD activity increased in those performing walking/square dancing, and 4-HNE levels decreased in those involved in taiji/yoga. Oxidative stress marker levels also improved in those involved in walking/square dancing or taiji/yoga groups as the exercise frequency increased. Thus, frequent participation in walking/square dancing or taiji/yoga effectively decreases hypertension-related oxidative stress biomarker levels.

## 1. Introduction

Hypertension is a common chronic condition that is the leading cause of morbidity and mortality globally [1, 2]. Population aging presents a tremendous challenge for China's hypertension control. The United Nations estimated that individuals aged ≥60 constituted 16.2% of the total Chinese population in 2017, and they predicted the percentage would increase to 35.1% by 2050 [3]. In recent years, there has been growing evidence that oxidative stress might be associated with the pathogenesis of hypertension, particularly in middle-aged and elderly people [2, 4].

Oxidative stress results from a systemic imbalance between reactive oxygen species (ROS) production and antioxidant capacity [5]. If ROS levels are not maintained at optimum levels, they can cause damage to cell membranes and macromolecules, including proteins, DNA, and RNA [2]. ROS levels may increase due to decreased antioxidant enzyme activity in individuals with hypertension, whereas decreasing oxidative stress may lower blood pressure [6]. Moreover, numerous studies have shown that ROS levels affect elderly people by enhancing lipid peroxidation and protein oxidation and altering antioxidant enzyme activity [7, 8]. An *α*,*β*-unsaturated hydroxyalkenal (4-hydroxynonenal [4-HNE]) produced during lipid peroxidation is generally considered to be a specific marker of oxidative stress [9]. Moreover, it is recognized as an important contributor to hypertension progression [10]. Previous studies have shown that 4-HNE levels are elevated in hypertensive rats, compared to normal animals, resulting in an accumulation of damaged proteins in the myocardium [11], whereas no accumulation of 4-HNE can counteract the deleterious effects of excessive oxidative stress in spontaneously hypertensive rats [12, 13]. Additionally, exercise training has a positive effect on spontaneously hypertensive rats by reducing cardiac lipid peroxidation and oxidative stress [12].

For middle-aged and elderly individuals, physical exercise is recommended as a means of reducing the risk of several chronic diseases [14]. Physical exercise can be beneficial to cardiovascular health because it can decrease vascular inflammation, inhibit oxidative stress, and improve plasma lipids, endothelial function, and coronary circulation [15–18]. Physical exercise may be important for preventing and treating hypertension and its associated pathologies. Although physical exercise can increase the production of ROS and free radicals, some studies have demonstrated that physical exercise benefits individuals with hypertension by improving their redox state [19]. Choosing the best type and intensity of physical exercise to help middle-aged and elderly people achieve a balance between exercise-related ROS production and antioxidant induction is important. However, few studies have described hypertension pathogenesis, in middle-aged and elderly individuals, relative to the oxidative stress effects of different types, frequencies, intensities, and durations of physical exercise [15, 17].

In China, essential hypertension patients are mostly diagnosed and treated in community hospitals. According to the baseline survey from the nationwide China Health and Retirement Longitudinal Study (CHARLS), hypertension had a higher underdiagnosis rate among middle-aged and elderly Chinese in community [20]. Therefore, it is valuable to develop a community-based study on prevention and treatment of hypertension. In addition, the implementation of health education and promotion has increased awareness of health and exercise among the middle-aged and elderly Chinese population, who are increasingly engaged in physical exercises such as walking, square dancing, taiji, yoga, running, biking, climbing, and ball sports [21]. Epidemiological studies also have shown that regular physical exercise performed at low to moderate intensity per week is more suitable for middle-aged and elderly people [22, 23]. Therefore, in the current study, we observed Chinese community residents who were participating in five types of physical exercise a week (walking/square dancing; taiji/yoga; running/biking/climbing; ball sports; gym workouts, including swimming). The study aimed to investigate the effect of different types and frequencies of physical exercises on SBP, DBP, and clinical characteristics and evaluated their levels of oxidative stress markers in the pathogenesis of essential hypertension, including superoxide dismutase (SOD) activity (a primary detoxification enzyme), malondialdehyde (MDA) levels, and 4-HNE (lipid peroxidation marker) levels.

## 2. Methods

### 2.1. Study Population

A community-based cross-sectional study was approved by the Ethics Committee of Bengbu Medical College and was conducted from June to August 2015 in Bengbu, China. Written informed consent was obtained from all participants before survey administration. According to stratified and random sampling methods, we aimed to recruit 4,000 participants who had resided for more than one year in Longzihu district of Bengbu, which include 7 subdistricts (32 communities); we randomly selected 125 potential participants in each community. Our research team visited each community and held a free health checkup camp in the morning for the consented participants. Their height, weight, and seated blood pressure were measured, and peripheral venous blood samples were collected at approximately the same time of day to minimize diurnal variation. Then, we scheduled a face-to-face 30-minute questionnaire interview at the home of each participant in the following two days after the checkup with trained interviewers using a structured questionnaire on an iPad device. For the home interview, we asked questions about the age, gender, education, marital status, income, life styles, and physical exercise information. Individuals were considered hypertensive if they met any of the following conditions: (1) had ever been told by a doctor or healthcare professional that he or she had hypertension, (2) self-reported prescribed antihypertensive medication use, or (3) diagnosed with systolic blood pressure (SBP) ≥140 mmHg and diastolic blood pressure (DBP) ≥90 mmHg [24].

**Figure 1:**
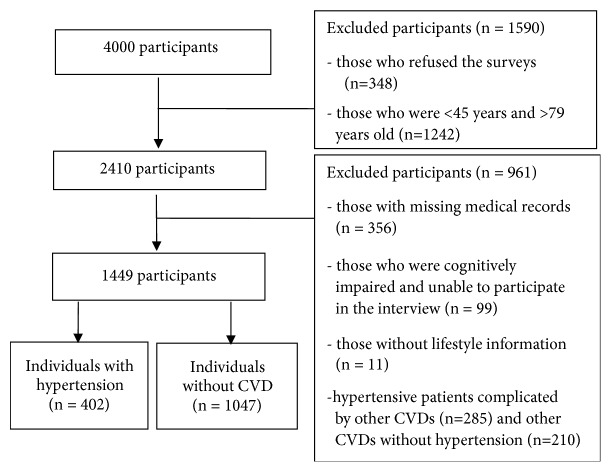
Flowchart of participant selection process.

Data were initially obtained from a total of 3,652 participates, due to 348 individuals' refusal to participate. Since this current study focuses on middle-aged and elderly community residents (age 45-79 years), we excluded 1,242 subjects who were younger than 45 years and older than 79 years. Among the remaining 2,410 potential study participants, we excluded subjects who did not undergo our onsite medical checkup operated by our research team (*n* = 356). We excluded subjects who were hypertensive patients complicated by other cardiovascular diseases (CVDs) (*n* = 285) and other CVDs without hypertension (*n* = 210). We also excluded subjects who could not participate in the 30-minute face-to-face interview due to serious cognitive impairment (*n* = 99) and who failed to provide physical exercise information in the questionnaire survey (*n* = 11). Lastly, a total of 402 individuals with essential hypertension and 1047 individuals without cardiovascular disease (CVD) were selected for this study. A detailed flowchart of the patient inclusion criteria is provided in Figure 1.

### 2.2. Measurement of Blood Pressure

Trained, certified staff took the blood pressure measurements. The seated blood pressure was measured three times with 5 min intervals between measurements in the morning. All SBP and DBP measurements were performed on the left arm using the auscultatory method following the recommendations of the American Heart Association [25]. To ensure the accuracy of the measurements, the blood pressure values were considered the mean of three measurements [26]. All participants were instructed to avoid physical exercise and alcoholic drinks for 24 h. They were also instructed not to ingest coffee, tea, caffeinated soft drinks, or other stimulants thereafter. Smokers were instructed not to smoke before testing sessions, and all patients continued to take their regular medication on experimental days [27].

### 2.3. Measurement of Physical Exercise

According to the international physical activity questionnaire short form [28], all participants answered interviewer-administered multichoice questions on physical exercise (PE) with the following questions: The first question is “Do you engage in any kind of physical exercise on a regular basis?” If someone answered “no” to the question, his or her frequency will be zero, and the following two questions will be skipped. If participants answered “yes” to the question, they will be asked “What kind of physical exercise do you do regularly?” We classified the types of exercise into five categories by intensity from low to high: PE1: walking/square dancing; PE2: taiji/yoga; PE3: running/biking/climbing; PE4: ball sports; PE5: gym workout including swimming [29]. Participants could select one or more of the options above. The third question is “For each physical exercise above, how many times a week do you exercise regularly?” Frequency of physical exercise was classified into three categories: low levels of physical exercise (0 times/week); moderate levels of physical exercise (1-3 times/week); high levels of physical exercise (4-7 times/week) [30].

### 2.4. Covariate Data

Each participant was asked for information about potential confounding factors, including demographic factors, socioeconomic status, and cardiovascular health-related lifestyle factors; family history; and current health condition. We considered the following as potential confounding variables: age, sex, education level (<9 years, 9-12 years, >12 years), marital status (married, widowed, divorced), income (<2000 RMB/month, 2000-3000 RMB/month, 3000-4000 RMB/month, >4000 RMB/month), smoking status, alcohol consumption, and fruit consumption.

### 2.5. Clinical Characteristics Measurements

After the participants had fasted for 8~12 h, their height, weight, blood pressure, and heart rate (HR) were measured; peripheral venous blood samples were collected in the morning at approximately the same time of day to minimize diurnal variation [31]. Each participant provided 5 ml fasting venous blood samples that were divided into two tubes. One part was allowed to clot and the serum was separated within 30 min, and the other part was transferred to an ethylenediaminetetraacetic acid tube and was centrifuged at 1509 x g for 30 min. Both serum and plasma were stored at -80°C until analyzed. The levels of glycosylated hemoglobin (HbA1c), total cholesterol (TC), and triglycerides (TG) were analyzed by routine blood and biochemistry tests. The ethics committee of the Centers for Disease Control and Prevention of Bengbu approved the sample collection for this study. Additionally, body mass index (BMI) was calculated according to the following formula: BMI = weight (kg)/height (m)^2^.

### 2.6. Laboratory Analysis

Plasma samples were evaluated for oxidative stress markers: superoxide dismutase (SOD) activity, malondialdehyde (MDA), and 4-hydroxynonenal (4-HNE) content via spectrophotometry. Colorimetry was conducted to determine the activity of SOD and concentration of MDA. The SOD activity and MDA content levels were measured spectrophotometrically at wavelengths of 550 and 532 nm using colorimetric assay kits (Nanjing Jiancheng Bioengineering Institute, Nanjing, China), according to the manufacturer's instructions [32]. The level of 4-HNE in human plasma was measured using an ELISA kit (Elabscience Biotechnology, Wuhan, China), according to the manufacturer's instructions [9].

### 2.7. Statistical Analysis

Descriptive statistics of demographic and clinical characteristics were provided. Specifically, we provided the mean and standard deviation (SD) for continuous variables and proportions for categorical variables. Differences between individuals with hypertension and individuals without CVD were examined using the *t*-test for continuous variables and Pearson *x*^2^ test for categorical variables. Differences among three frequencies of physical exercise were examined using one-way analysis of variance with Tukey's post hoc multiple comparison test. Statistical analyses were performed using SPSS software (version 24.0, IBM Corp, Armonk, NY, USA). Values were considered statistically significant at *p* < 0.05.

In order to evaluate whether markers of oxidative stress in individuals with hypertension and individuals without CVD had the same model for different PEs, multilevel linear regression models were conducted in R 3.4.3, while adjusting for the hierarchical data structure of the three levels: individual, community, and subdistrict. We assumed that the environmental exposures, socioeconomic status, and behavioral patterns were shared by individuals within the same community but not across communities. Similarly, these factors were assumed to be shared by individuals within the same subdistrict but not across subdistricts. In order to account for the individual dependence between each community as well as community dependence between subdistricts, two sets of random intercepts were included and the model was as follows:

(1) $$\begin{array}{c} Y_{ijk}=\gamma_{0}+\overset{8}{\underset{m=1}{\sum }}\gamma_{m}X_{m,ijk}+\overset{6}{\underset{s=1}{\sum }}\beta_{s}PE_{s,ijk}+v_{0,jk}+b_{0,k} \end{array}$$

where Y_ijk_ denotes the level of biomarkers of hypertension (SBP, DBP, MOD, SOD, 4-HNE) for the ith individual in the jth community within the kth subdistrict, PE_s,ijk_ (*s* = 1,…, 5) denotes the status of the sth type of physical exercise for the ith individual within jth community under the kth subdistrict, and X_m,ijk_ (*m* = 1,…, 9), respectively, represents one of nine confounders (age, gender, education, marital status, income, smoking, alcohol, fruit, and BMI). Finally, *ν*_0,jk_ represents the random intercept for the jth community under the kth subdistrict, and b_0,k_ represents the random intercept for kth subdistrict. We assumed that *ν*_0,jk_, b_0,k_ are independent random variables from normal distributions with mean 0.

## 3. Results

### 3.1. Baseline Characteristics of the Study Population

The demographic characteristics of all subjects are shown in Table 1. At the time of the study visit, there were no significant differences in the education, income, and alcohol consumption. However, age, gender, marital status, smoking status, and fruit consumption were significantly different.

### 3.2. Clinical Characteristics

The clinical characteristics of the study participants are shown in Table 2. There were no significant differences in terms of DBP values or TC levels. However, the averages of the BMI, SBP, and HR and the levels of HbA1c, TG, MDA, and 4-HNE were all significantly higher in individuals with hypertension than in those without CVD (*p* < 0.05). We also found that SOD was significantly lower in individuals with hypertension than in those without CVD.

### 3.3. Association between Physical Exercises and Blood Pressure

Table 3 shows the association between the five PE types and blood pressure indexes (SBP, DBP). For PE1, we observed a significant decrease in SBP with high frequency exercise in individuals with hypertension and individuals without CVD, while DBP was decreased only in individuals with hypertension. For PE2, we only observed a significant decrease in SBP with high frequency exercise in both groups. For PE3, we only found a significant decrease in SBP with moderate frequency exercise in individuals without CVD.

### 3.4. Association between Physical Exercises and Clinical Characteristics

Table 4 shows the association between types of PE and clinical characteristics (BMI, HR, HbA1c, TG). For PE1, we observed a significant decrease in HbA1c with high frequency exercise in individuals with hypertension and individuals without CVD, while BMI was decreased only in individuals without CVD. For PE2, we only observed a significant decrease in HR with high frequency exercise in individuals with hypertension. For PE3, we found a significant decrease in TG with moderate frequency exercise in individuals with hypertension and individuals without CVD and also found a significant decrease in BMI with moderate frequency exercise in individuals without CVD.

### 3.5. Association between Types of Physical Exercise and Oxidative Stress Markers

**Figure 2:**
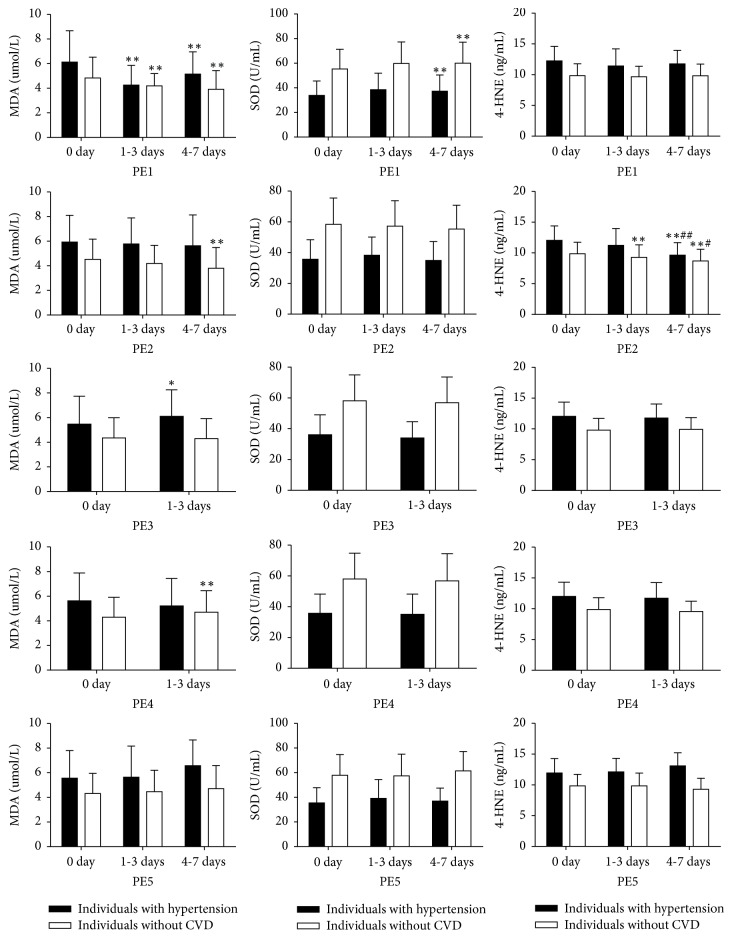
Relationships between the five types of PE and oxidative stress markers MDA: malondialdehyde; SOD: superoxide dismutase; 4-HNE: 4-hydroxynonenal; PE1: walking/square dancing; PE2: taiji/yoga; PE3: running/biking/climbing; PE4: ball sports; PE5: gym workout including swimming. Data are presented as means ± SD. ^*∗*^*p* < 0.05 versus 0 days, ^*∗∗*^*p* < 0.01 versus 0 days; ^#^*p* < 0.05 versus 4-7 days, ^##^*p* < 0.01 versus 4-7 days.

The relationships between each type of physical exercise and oxidative stress markers (MDA, SOD, 4-HNE) in individuals with hypertension and individuals without CVD are shown in Figure 2. PE1 was not statistically significantly associated with 4-HNE, but it was significantly associated with MDA levels and SOD activities in both participant types (*p* < 0.01). MDA levels were reduced and the SOD activities were increased in both participant groups participating in a high frequency of PE1. For PE2, a significant decrease in MDA levels was associated with high frequency of exercise participation for individuals without CVD. Increased frequency of PE2 participation were also associated with significant 4-HNE level decreases in both participant groups (*p* < 0.01). Moderate frequency of PE3 increased MDA levels in participants with hypertension, but not in those without CVD (*p* < 0.05).

### 3.6. Multilevel Linear Regression Model

Multilevel linear regression analysis showed an association between the five types of PE and oxidative stress markers in the two study groups after adjusting for age, sex, education, marital status, smoking, alcohol drinking, fruit consumption, BMI, SBP, DBP, HR, HbA1c, TG, and TC. MDA levels were decreased in PE1, SOD levels were increased in PE1, and 4-HNE levels were decreased in PE2 in individuals with hypertension, and this difference was statistically significant. In individuals without CVD, MDA levels were decreased in PE1, PE2, and PE5, SOD levels were increased in PE1 and PE3, and 4-HNE levels were decreased in PE2; this difference was statistically significant (Table 5).

## 4. Discussion

Previous studies suggested that physical exercise was an effective intervention in the prevention and treatment of hypertension and cardiovascular disease via a reduction in oxidative stress [19]. The aim of this study was to investigate the effect of the type and frequency of physical exercise on biomarkers of oxidative stress with hypertension in middle-aged and elderly. We found that walking/square dancing and taiji/yoga had improved oxidative stress markers in middle-aged and elderly individuals with hypertension as the frequency of exercise increased. To our knowledge, this study is one of the first to examine the relationship between the type and frequency of physical exercise and oxidative stress on hypertension using a community-based cross-sectional study in China.

Since 1983, the World Health Organization recommended the use of nonpharmacological approaches for primary and adjunctive treatment for hypertension [33]. Regular physical exercise was recommended for the maintenance of optimal health and prevention or management of hypertension [34]. Numerous epidemiologic and experimental studies have shown that physical exercise is an important intervention for cardiovascular risk factors [35–38]. A meta-analysis concluded that aerobic exercise training lowered blood pressure by 5-7 mmHg in adults with hypertension, while dynamic resistance training lowered blood pressure by 2-3 mmHg [39]. Exercising as little as 1 day per week is as effective as pharmacotherapy (or even more so) for reducing all-cause mortality associated with hypertension [40]. However, there is little information on the different types of physical exercise that effectively attenuate hypertension among the middle-aged and elderly community residents in China. In our study, we divided types of physical exercise into five categories, according to the traditional Chinese regular exercise in middle-aged and elderly individuals [41–43]. As we expect, the principal results of the present study also showed a link between physical exercise and blood pressure. Among the individuals who participated in walking/square dancing, we observed a significant decrease in SBP with the high frequency of exercise in both study groups, while DBP was decreased only in individuals with hypertension. Among the individuals who participated in taiji/yoga, we only observed a significant decrease in SBP with high frequency of exercise in the two study groups. Among the individuals who participated in biking/climbing, we only found a significant decrease in SBP with a moderate frequency exercise in individuals without CVD. These results suggested that walking/square dancing and taiji/yoga may have an effective antihypertensive effect. This is in agreement with Wallace [44]; type of physical exercise needs to be considered for the treatment of hypertension. Aerobic and resistance exercise have been recognized as effective in the prevention of hypertension [45, 46]; however, their role in the treatment of hypertension is not clear.

In our study, we also further explored the association between type and frequency of physical exercise and some clinical characteristics. Long-term regular physical exercise has been shown to noticeably reduce blood pressure and remarkably attenuate symptoms of hypertension [47]. For walking/square dancing, we found a significant decrease in HbA1c with high frequency in both study groups, while BMI was decreased only in individuals without CVD. For running/biking/climbing, we found a significant decrease in TG with moderate frequency exercise in two groups. We also observed a significant decrease in HR with high frequency of taiji/yoga in individuals with hypertension. These results suggested that those exercises have a favorable effect on cardiovascular health, especially for the prevention or treatment of hypertension. Engaging in regular physical exercise enhances structural, functional, and biochemical characteristics of the cardiovascular system and improves cardiovascular risk factors among individuals with normal blood pressure, as well as those with prehypertension or hypertension [48]. Furthermore, regular physical exercise has been shown to inhibit oxidative stress and inflammatory markers, to improve VO_2_ peak and aerobic capacity, and to favorably modulate plasma superoxide dismutase activity and endothelial function [16, 18].

Oxidative stress plays a key role in the pathophysiology of hypertension [19, 49]. The importance of redox imbalance in hypertension has also been demonstrated in many population-based studies. Sustained accumulation of free radicals due to disrupted redox homeostasis negatively affects vascular function, thus contributing to the onset and progression of hypertension [12]. Clinical studies of patients with essential hypertension demonstrated that SBP and DBP correlate positively with biomarkers of oxidative stress and negatively with antioxidant levels. Decreased antioxidant activity (SOD) and increased levels of lipid peroxidation (MDA) might contribute to oxidative stress in human hypertension [1, 50, 51]. Naregal et al. found that the levels of plasma MDA were significantly raised and SOD antioxidant activity was decreased in elderly individuals with hypertension compared to healthy controls [2]. Additionally, some studies have demonstrated that exercise may be able to attenuate oxidative damage and increase the clearance of reactive aldehydes in patients with hypertension [2, 12]. Considering the effect of physical exercise on oxidative stress, we described different types and frequencies of physical exercise in middle-aged and elderly individuals with hypertension.

In the present study, we applied multilevel linear regression model to further evaluate whether markers of oxidative stress in individuals with hypertension and individuals without CVD had the same model for different physical exercises. Because of environmental exposures, socioeconomic status, and behavioral patterns shared by individuals within the same community but not across communities, we accounted for hierarchical data structure of three levels: individual, community, and subdistrict. Our findings demonstrated that the MDA levels were decreased and the SOD levels were increased in individuals with hypertension who participated in walking/square dancing. In individuals without CVD, the MDA levels were decreased in individuals who participated in walking/square dancing, taiji/yoga, and gym workout including swimming, and the SOD levels were increased in individuals who participated in walking/square dancing and running/biking/climbing. Interestingly, this study also showed that running/biking/climbing and gym workout including swimming had protective effects against oxidative stress damage only in individuals without CVD. In addition, with the increase of exercise frequency, the oxidative stress markers were improved in individuals who participated in walking/square dancing and taiji/yoga. These results mainly suggested that individuals with hypertension should engage in more walking/square dancing and taiji/yoga, especially at a frequency of 4-7 days per week. A similar explanation had been offered by Rodrigues-Krause et al.: dance might be a potential exercise intervention for improving cardiorespiratory fitness and consequent cardiovascular risk associated with aging [18]. Regular dance training could have a positive effect on body fat, VO_2_ peak, blood total antioxidant capacity, and muscle damage markers [46, 52]. Furthermore, our study also found that 4-HNE levels were reduced only in individuals who participated in taiji/yoga in both groups.

4-HNE is the major cytotoxic aldehyde generated during lipid peroxidation [53]. Campos et al. proved that 4-HNE was increased through lipid peroxidation during hypertension-induced compensated cardiac hypertrophy [12]. In the present study, we found that yoga and taiji could reduce 4-HNE protein levels both in patients with hypertension and in individuals without CVD. Similarly, Tolahunase et al. reported that the levels of ROS and the rate of cellular aging in an apparently healthy population were significantly reduced after 12 weeks of yoga intervention [54]. It may explain why taiji/yoga exercises inhibited the expression of 4-HNE in middle-aged and elderly. However, the underlying mechanisms need to be further examined.

There were several limitations in this study that are important to note. First, our cross-sectional design is subject to the problems of (1) not being able to discern the direction of causation, (2) potential unadjusted individual-level confounders, and (3) ignoring participants' earlier life influences that may have impacted their risk of adverse CVD outcomes. Second, this study only observed types and frequencies of exercise and did not include intensities and durations. However, this study firstly reported the relation of some types of physical exercise and oxidative stress in hypertension in middle-aged and elderly individuals; we plan to design the duration and intensity in next study. Lastly, in spite of an advanced sample size estimate, the risk factor analysis was not considered and sample size of subgroups was low. This may affect the study results. Future studies that are community-based and longitudinal in design will be necessary to identify additional oxidative stress markers of hypertension and endurance exercise in middle-aged and elderly.

## 5. Conclusion

The results of this study indicate that the walking, square dancing, taiji, and yoga are effective at decreasing the levels of oxidative stress biomarkers in individuals with hypertension, particularly as the frequency of physical exercise increases. This underscores the importance of including walking, square dancing, taiji, and yoga into local community hypertension prevention programs. We look forward to thoroughly exploring the role of physical exercise and its association with oxidative stress in middle-aged and elderly individuals with hypertension.

## Figures and Tables

**Table 1:** Demographics of the study participants.

Variables	Individuals with hypertension (*n* = 402)	Individuals without CVD (*n* = 1047)	*t*/*x*^2^	*p*-value
Age, years	63.82 ± 10.42	57.88 ± 10.90	9.39	0.001
Men, n (%)	192 (47.8)	418 (39.9)	7.32	0.007
Years of Education			2.04	0.361
<9 years	137(34.1%)	326(31.1%)		
9-12 years	233(58.0%)	649(62.0%)		
>12 years	32(8.0%)	72(6.9%)		
Marital status			8.71	0.033
Married	325(80.8%)	899(85.9%)		
Widower	62(15.4%)	104(9.9%)		
Divorced	15(3.7%)	44(4.2%)		
Income (RMB/month)			4.69	0.196
<2000	80(19.9%)	161(15.4%)		
2000-3000	128(31.8%)	357(34.1%)		
3000-4000	141(35.1%)	372(35.5%)		
>4000	53(13.2%)	157(15.0%)		
Smoking status, n (%)			13.32	0.001
No	195(48.5%)	873(83.4%)		
Yes	207(51.5%)	174(16.6%)		
Alcohol consumption, n (%)			1.47	0.225
No	139(34.6%)	398(38.0%)		
Yes	263(65.4%)	649(62.0%)		
Fruit consumption, n (%)			11.86	0.001
No	139(34.6%)	267(25.5%)		
Yes	263(65.4%)	780(74.5%)		

Data are presented as mean ± SD.

**Table 2:** Clinical characteristics of the study participants.

Variables	Individuals with hypertension (*n* = 402)	Individuals without CVD (*n* = 1047)	*t*	*p*-value
Age, years	63.82 ± 10.42	57.88 ± 10.90	9.39	0.001
Men, n (%)	192 (47.8)	418 (39.9)	7.32	0.007
BMI (kg/m^2^)	25.72 ± 4.83	24.13 ± 3.68	6.47	0.001
SBP (mmHg)	141.02 ± 16.03	126.68 ± 17.46	14.87	0.001
DBP (mmHg)	81.03 ± 8.57	80.58 ± 9.42	1.15	0.249
HR (beats/min)	77.50 ± 7.35	72.52 ± 5.29	14.31	0.001
HbA1c (%)	5.31 ± 1.18	5.17 ± 1.15	2.02	0.044
TC (mmol/L)	5.13 ± 0.90	5.03 ± 0.97	1.78	0.076
TG (mmol/L)	1.95 ± 1.66	1.59 ± 1.33	4.40	0.001
MDA (nmol/mL)	5.59 ± 2.26	4.34 ± 1.64	10.13	0.001
SOD (U/mL)	35.69 ± 12.53	57.88 ± 16.86	27.26	0.001
4-HNE	11.98 ± 2.31	9.83 ± 1.89	16.65	0.001

BMI: body mass index; SBP: systolic blood pressure; DBP: diastolic blood pressure; HR: heart rate; HbA1c: glycosylated hemoglobin; TC: total cholesterol; TG: triglycerides; MDA: malondialdehyde; SOD: superoxide dismutase; 4-HNE: 4-hydroxynonenal. Data are presented as means ± SD.

**Table 3:** Relationships between blood pressure and the five types of PE.

Type of PE	Frequency	Individuals with hypertension (*n* = 402)		Individuals without CVD (*n* = 1047)	
		SBP (mmHg)	DBP (mmHg)	SBP (mmHg)	DBP (mmHg)
PE1	0 days	143.72 ± 14.98	83.83 ± 8.62	129.88 ± 17.58	81.18 ± 9.09
	1-3 days	141.15 ± 16.11	80.00 ± 7.85	126.17 ± 19.13	81.89 ± 7.84
	4-7 days	139.70 ± 17.02^*∗*^	79.14 ± 8.55^*∗*^	126.24 ± 17.19^*∗*^	80.68±9.18
PE2	0 days	145.36 ± 17.68	83.27 ± 12.21	129.22 ± 19.51	82.75 ± 9.90
	1-3 days	142.32 ± 15.10	83.03 ± 8.15	127.77 ± 17.29	80.88 ± 9.02
	4-7 days	137.44 ± 11.31^*∗*^	81.00 ± 8.69	125.64 ± 14.96^*∗*^	80.48 ± 8.89
PE3	0 days	128.01 ± 17.50	81.73 ± 8.94	142.84 ± 16.36	80.80 ± 8.99
	1-3 days	127.17 ± 17.76	80.46 ± 8.39	137.08 ± 13.94^*∗*^	81.77 ± 9.44
PE4	0 days	141.79 ± 16.18	81.50 ± 8.91	127.70 ± 17.51	80.84 ± 8.9
	1-3 days	141.44 ± 15.31	81.44 ± 8.27	129.09 ± 17.84	81.89 ± 10.11
PE5	0 days	144.48 ± 16.66	84.08 ± 10.42	128.02 ± 17.76	81.03 ± 8.94
	1-3 days	142.50 ± 19.67	83.90 ± 8.40	126.97 ± 15.72	80.22 ± 9.76
	4-7 days	141.30 ± 15.91	81.25 ± 8.72	125.10 ± 16.39	81.75 ± 11.56

PE: physical exercise; CVD: cardiovascular disease; SBP: systolic blood pressure; DBP: diastolic blood pressure; PE1: walking/square dancing; PE2: taiji/yoga; PE3: running/biking/climbing; PE4: ball sports; PE5: gym workout including swimming. Data are presented as means ± SD.

^*∗*^ *p* < 0.05 versus 0 days.

**Table 4:** Relationships between clinical characteristics and the five types of PE.

Type of PE	Frequency	Individuals with hypertension (*n* = 402)				Individuals without CVD (*n* = 1047)			
		BMI	HR	HbA1c	TG	BMI	HR	HbA1c	TG
PE1	0 days	25.70 ± 4.19	77.56 ± 7.51	5.71 ± 1.41	2.02 ± 0.83	24.94 ± 2.79	72.75 ± 5.55	5.75 ± 1.08	1.53 ± 0.97
	1-3 days	25.65 ± 5.35	77.10 ± 6.06	5.29 ± 1.18	1.70 ± 0.81	23.57 ± 2.38	72.35 ± 4.75	5.23 ± 1.22	1.64 ± 1.07
	4-7 days	25.19 ± 3.51	77.47 ± 7.33	5.09 ± 1.15^*∗*^	1.89 ± 0.72	22.19 ± 2.54^*∗*^	72.31 ± 5.11	4.35 ± 1.04^*∗*^	1.62 ± 1.59
PE2	0 days	26.12 ± 3.39	79.13 ± 6.63	5.32 ± 1.22	2.06 ± 1.61	24.21 ± 4.07	72.45 ± 5.41	5.18 ± 1.15	1.60 ± 1.43
	1-3 days	25.59 ± 5.11	77.91 ± 7.33	5.43 ± 1.33	1.93 ± 1.65	23.99 ± 3.52	73.36 ± 4.59	5.11 ± 1.11	1.56 ± 0.60
	4-7 days	24.82 ± 3.50	77.08 ± 7.47^*∗*^	5.21 ± 0.93	1.87 ± 0.95	23.62 ± 4.09	72.38 ± 4.94	5.16 ± 1.18	1.49 ± 0.91
PE3	0 days	25.74 ± 4.91	77.31 ± 7.32	5.28 ± 1.16	1.99 ± 0.72	24.99 ± 2.68	73.01 ± 5.42	5.14 ± 1.13	1.61 ± 0.47
	1-3 days	25.22 ± 4.11	78.28 ± 7.43	5.44 ± 1.27	1.52 ± 0.75^*∗*^	22.02 ± 2.48^*∗*^	72.41 ± 5.26	5.31 ± 1.21	1.49 ± 0.44^*∗*^
PE4	0 days	25.66 ± 4.79	77.66 ± 7.47	5.29 ± 1.15	1.97 ± 1.74	24.35 ± 3.83	72.45 ± 5.22	5.19 ± 1.16	1.59 ± 1.37
	1-3 days	25.53 ± 4.53	76.00 ± 5.89	5.51 ± 1.43	1.86 ± 1.26	23.95 ± 3.62	72.95 ± 5.78	5.01 ± 1.02	1.51 ± 0.88
PE5	0 days	26.10 ± 3.81	77.64 ± 7.32	5.31 ± 1.18	1.92 ± 1.65	24.12 ± 3.24	72.52 ± 5.22	5.17 ± 1.15	1.60 ± 1.38
	1-3 days	25.63 ± 4.82	76.04 ± 7.85	5.30 ± 1.25	2.20 ± 1.81	24.06 ± 3.69	72.42 ± 5.70	5.18 ± 1.16	1.41 ± 0.80
	4-7 days	25.73 ± 4.90	75.80 ± 6.92	5.27 ± 0.79	2.25 ± 1.36	23.35 ± 3.26	72.50 ± 6.56	5.41 ± 1.28	1.56 ± 0.57

PE: physical exercise; CVD: cardiovascular disease; BMI: body mass index; HR: heart rate; HbA1c: glycosylated hemoglobin; TG: triglycerides; PE1: walking/square dancing; PE2: taiji/yoga; PE3: running/biking/climbing; PE4: ball sports; PE5: gym workout including swimming. Data are presented as means ± SD.

^*∗*^ *p* < 0.05 versus 0 days.

**Table 5:** Multilevel linear regression on PE and oxidative stress markers.

Variable	MDA		SOD		4-HNE	
	*β*	*p*	*β*	*p*	*β*	*p*
Individuals with hypertension						
PE1	-0.461	0.001	2.385	0.001	-0.234	0.075
PE2	-0.247	0.106	-0.719	0.405	-1.228	0.001
PE3	-0.193	0.511	-1.700	0.306	-0.287	0.352
PE4	-0.200	0.619	1.087	0.633	-0.268	0.526
PE5	0.152	0.262	0.443	0.798	0.293	0.363
Individuals without CVD						
PE1	-0.461	0.001	2.269	0.001	-0.030	0.648
PE2	-0.501	0.001	-1.222	0.131	-0.563	0.001
PE3	-0.007	0.959	2.088	0.012	0.129	0.428
PE4	0.218	0.195	-1.603	0.366	-0.246	0.215
PE5	-0.615	0.045	0.827	0.563	-0.092	0.564

MDA: malondialdehyde; SOD: superoxide dismutase; 4-HNE: 4-hydroxynonenal; PE1: walking/square dancing; PE2: taiji/yoga; PE3: running/biking/climbing; PE4: ball sports; PE5: gym workout including swimming.

*β*:regression coefficient.
